# A study on musculoskeletal complaints and working postures in pathology specialists in Iran

**DOI:** 10.1186/s12891-021-04870-w

**Published:** 2021-12-03

**Authors:** Ehsan Rafeemanesh, Alireza Khooei, Shabnam Niroumand, Tina Shirzadeh

**Affiliations:** 1 Department of Occupational Medicine, Medical Toxicology Research Center, Faculty of Medicine, Mashhad University of Medical Sciences, Mashhad, Iran; 2 Department of Pathology, Faculty of Medicine, Mashhad University of Medical Sciences, Mashhad, Iran; 3 Faculty of Medicine, Mashhad University of Medical Sciences, Mashhad, Iran; 4 Occupational Medicine Department, Faculty of Medicine, Mashhad University of Medical Sciences, Azadi Square. Mashhad, Pardis, Iran

**Keywords:** Work-related musculoskeletal disorders (WMSDs), Pathologist, Nordic questionnaire, Posture, Rapid upper limb assessment (RULA)

## Abstract

**Background:**

Musculoskeletal disorders (MSDs) are one of the most common causes of occupational injuries and disabilities among health care workers. This study investigates the relationship between musculoskeletal complaints and pathologist postures in laboratories.

**Method:**

In this cross-sectional study, 40 pathologists were evaluated. MSDs in different body segments of the participants were evaluated by Nordic questionnaire. For postural analysis, 20 min film was recorded while using a microscope by subjects. Posture analysis was done by the Rapid Upper Limb Assessment (RULA) method and their repetitive movements were scored. The data was analyzed by SPSS Version 11.5.

**Results:**

The mean age and duration of employment of subjects was 36.57 ± 7.54 years and 6.50 ± 6.30 years, respectively. Most MSDs were found in neck (65%), wrist (57.5%), upper back (50%) and lower back (47.5%). The mean RULA grand score was higher in participants with upper back and shoulder pain. A statistically significant relationship was found between the mean RULA grand, the upper back pain (*P* = 0.02) and the wrist pain (*P* = 0.003), as well as between the mean RULA B, the neck pain (*P* = 0.02) and the lower back pain (*P* = 0.05). The results showed a significant relationship between mean weekly working hours and tight (*P* < 0.001), wrist (*p* = 0.01) and ankle (*P* = 0.008).

**Conclusion:**

This study revealed high prevalence of MSDs among the pathologists. Therefore, performing ergonomic corrective actions is essential in order to improve their physical conditions at work.

## Background

As each person spends more than one third of own life in the working environment, the hazardous factors of the workplace, including ergonomic factors, affect the individual’s health. Work-related musculoskeletal disorders (WMSDs) reduce the power and quality of work, increase the treatment costs and lost work time and result early disability [1].

The National Institute for Occupational Safety and Health (NIOSH) classifies the musculoskeletal discomforts in the second rank of work-related illnesses and complications based on their importance in terms of prevalence and severity [2]. Biomechanical risk factors which causing or exacerbating MSDs includes repetitive movements, inappropriate postures, frequent carrying heavy loads, excessive force, delicate and repetitive works and vibration, among which the inappropriate posture is the most common and important risk factor [3]. The WMSDs constitute a considerable part of MSDs and include complications from work activities that occur mainly due to repetitive muscle activity and repeated mechanical pressure [4]. Annually the Occupational Safety and Health Administration (OSHA) reports 200 cases of WMSDs compensation claims, including carpal tunnel syndrome (CTS), tendonitis, tenosynovitis, epicondylitis, and low back pain [5]. The main risk factors of MSDs are high physical activity, poor body fitness, repetitive movements, and pressure on the body due to local contact with chemicals. In all cases, contact duration and magnitude of these factors are considerably significant [6]. Individual characteristics, such as height, gender, race, socioeconomic factors, and coping ability to stress, affect the development of these disorders [7].

Evidence suggests a significant relationship between awkward working postures and pain and musculoskeletal system damage. The awkward working posture is defined as a significant deviation from the natural posture. Some examples of these awkward working postures include back stretching, wresting, overhead working, wrist rotating, knee ling, forward and backward bending and squatting [8].

The result of the study of MacDonald K and King D [9] on echo cardiographers specialists showed that pain is common (44%) in these specialists and it is significantly associated with gender, and also affects job performance for over one-third of pain sufferers.

Study on Correlation between risk factors and MSD among classical musicians showed.

the biomechanical risk factors that predict playing related musculoskeletal disorders are mainly associated with the upper limbs [10].

Pathologists are at higher risk of developing musculoskeletal symptoms due to repetitive movements, long-term work in unceasing static conditions and prolonged microscope use. The prevalence of musculoskeletal symptoms at least in one body segment among medical laboratory technicians have been reported to be between 72.5 and 92.3% [2]. In previous studies in Iran, the prevalence of symptoms of MSDs were assessed in pathologists and the most common complaints were pain in neck (33.3%) followed by shoulder (9.8%) and elbow (8.7%) [11, 12].

WMSDs can significantly increase direct and indirect medical costs by affecting pathologist’s occupational health and affect the pathologist’s accuracy judgment in diagnosing laboratory slides and samples. The main focus of most studies on laboratory personnel is on biological and chemical hazards and few evidences is available for the ergonomic hazards that eventually lead to musculoskeletal disorders. Considering the mentioned problems, this study aimed to evaluate the prevalence of MSDs symptoms in all pathologists of Mashhad University of medical sciences and assess the Relationship between musculoskeletal complaints and working posture scores.

## Methods

### Study population

This is a cross sectional study of all pathology specialists and residents in Mashhad University of Medical Sciences in 2018. Pathology specialists are the specialized medical doctor who assess the body tissues or fluids specimen in order to help other medical specialists to diagnose different disease. The purpose of the study was explained to participants and all who agreed to take part, provided oral informed consent. This study was done according to the approved research proposal number 950455 and Mashhad University Medical Sciences ethics committee in number IR.MUMS.fm. REC.1395.437 approved study method. Subjects with congenital musculoskeletal disorders or chronic rheumatologic diseases and those with a history of severe trauma were excluded from the study. From all 46 pathologists who worked in Mashhad University, 40 subjects had eligible criteria to involve in study.

### Instruments

The instrument used in this study to evaluate musckoloskeletal disorders in different body area was Nordic questionnaire, which was developed in Occupational Health Institute in Nordic countries to assess MSDs in 1987 and was validated in previous studies [13].

The Nordic questionnaire has been designed in four sections consisting of general questions, determining the complications and discomforts of the organs, detecting the status of the workplace leaving due to discomfort of the organs and discomfort details in eight parts of the neck, upper back, lower back, shoulder, femur, knee, wrist and ankle. Participants under the supervision of an occupational medicine resident or a trained expert completed the questionnaire.

Rapid upper limb assessment (RULA) was used to evaluate upper extremities MSD risk factors in pathologists. The RULA method was developed by two ergonomists in Nottingham University in England in 1993 named Dr. Lynn Mc Atamney and Professor E. Nigel Corlett [6]. This tool assesses the biomechanical and postural risk factors to a worker. In this study, a trained expert in occupational health and safety recorded a 20 min video from pathologists and their most repeated movements were extracted for analysis by RULA instrument. A single worksheet was used to analyze posture, exertion force and repetition of movements. The evaluator assigned the score to the postures of arm, forearm and wrist in section A in worksheet, and to the postures of neck, trunk and leg in section B. The Final RULA score was generated from the sum of the RULA *A* and RULA *B*. Then final RULA score categorized into four level of MSD risk as follows:

- Level 1 (final RULA score: 1–2): Negligible risk, acceptable postures
- Level 2 (final RULA score: 3–4): Low risk, further examination and probably the need for postural change
- Level 3 (final RULA score: 5–6): Medium risk, further examination and the need for postural change soon
- Level 4 (final RULA score: 7+): Very high risk, further examination and the need for postural change immediately.

### Data analysis

All quantitative variables were presented as mean and standard deviation (SD). Qualitative variables were reported as exact number and percent. For comparison between the means, the student’s t test or the Mann-Whitney U test was used after assessing the condition of normality by Kolmogorov-Smirnoff test. The chi-square test or Fisher’s exact test was used to evaluate the association between qualitative variable. The binomial logistic regression was then run to determine the factors significantly associated with MSDs in upper body areas in pathologists. Data were analyzed using SPSS (Statistical package for social science) version 11.5. In all calculations the statistically significance level was considered as < 0.05.

## Results

**Fig. 1:**
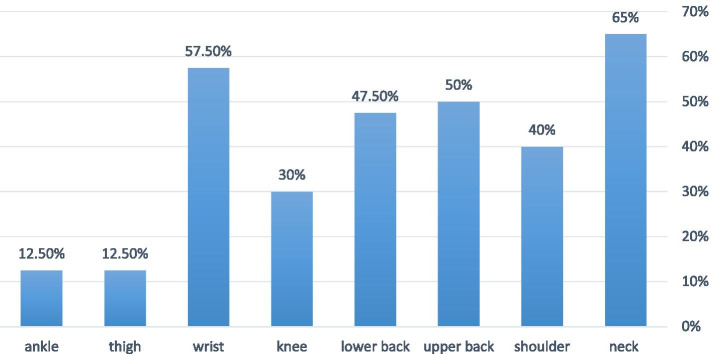
Prevalence of musculoskeletal disorder in study population during the past 12 months

Forty pathologists were enrolled in the study. The mean age of the subjects was 36.57 ± 7.54 years and the mean duration of occupation was 6.50 ± 6.30 years. According to the pathologist’s report, the mean weekly working hours was 45.15 ± 12.84. The mean height, weight and body mass index (BMI) of the participants were 165.75 ± 9.95 cm, 66.05 ± 12.25 kg and 23.83 ± 2.16 kg/m^2^ respectively. A summary of participant’s characteristics was presented in Table 1. As Fig. 1 shows most of the pathologists reported MSD symptoms in neck (65%), wrist (57.5%) and upper back (50%) and fewer of them had symptoms in lower limbs (12.5%). According to Table 2 the mean RULA A was higher in pathologists who report pain in their wrist (*P* value = 0.003) and the mean RULA B was statistically significant higher in participants with neck pain (*p* value = 0.02). Beside the RULA grand was statistically significant higher in person with upper back pain and shoulder pain, compared with the person without pain in these part of musculoskeletal system (*p* value = 0.02, *P* value = 0.02).

**Table 1:** Demographic characteristics of study population (*n* = 40)

Variables		Frequency	percentage
**gender**	Female	27	67.5
	Male	13	32.5
**BMI**	Normal	29	72.5
	Overweight/Obesity	11	27.5
**Dominant hand**	Right	35	87.5
	Left	5	12.5
**Smoking**	Yes	1	2.5
	No	39	97.5

**Table 2:** Relationship between musculoskeletal complaints and working posture scores

Organs		RULA grand Mean (SD)	RULA A Mean (SD)	RULA B Mean (SD)
**Neck pain**	Yes (*n* = 26)	5.38 (0.89)	5.56 (0.89)	4.80 (0.40)
	No (*n* = 14)	4.85 (1.02)	5.50 (0.65)	4.42 (0.64)
	*P* value	0.1	0.5	0.02
**Upper back pain**	Yes (*n* = 20)	5.55 (0.99)	5.58 (0.98)	4.80 (0.41)
	No (*n* = 20)	4.85 (0.81)	5.35 (0.48)	4.55 (0.60)
	*P* value	0.02	0.5	0.1
**Lower back pain**	Yes (*n* = 19)	5.15 (0.89)	5.57 (0.96)	5.57 (0.96)
	No (*n* = 21)	5.23 (1.04)	5.61 (0.66)	4.52 (0.60)
	*P* value	0.7	0.8	0.05
**Shoulder pain**	Yes (*n* = 16)	5.62 (1.08)	5.81 (0.98)	4.62 (0.61)
	No (*n* = 24)	4.91 (0.77)	5.45 (0.65)	4.70 (0.47)
	*P* value	0.02	0.1	0.6
**Thigh pain**	Yes (*n* = 5)	5.60 (0.89)	5.60 (0.84)	4.80 (0.44)
	No (*n* = 35)	5.14 (0.97)	5.60 (0.54)	4.65 (0.53)
	*P* value	0.3	1.00	0.5
**Knee pain**	Yes (*n* = 12)	4.91 (0.79)	5.41 (0.51)	4.75 (0.45)
	No (*n* = 28)	5.32 (1.02)	5.67 (0.90)	4.62 (0.55)
	*P* value	0.2	0.3	0.5
**Wrist pain**	Yes (*n* = 23)	5.30 (1.1)	5.91 (0.90)	4.60 (0.58)
	No (*n* = 17)	5.05 (0.74)	5.17 (0.39)	4.76 (0.43)
	*P* value	0.4	0.003	0.3
**Ankle pain**	Yes (*n* = 5)	4.60 (0.89)	5.60 (0.54)	4.40 (0.89)
	No (*n* = 35)	5.28 (0.95)	5.60 (0.84)	4.71 (0.45)
	*P* value	0.1	1.00	0.4

All *P* value were calculated with Mann Whitney U test

Generally, the RULA grand score was 3 or 4 in 8 (20.0%), 5 or 6 in 27 (67.5%) and 7 in 5 (12.5%) subjects. Most of the pathologists who reported pain in their neck (73.1%), upper back (65%), lower back (68.4%), shoulder (68.8%) and wrist (60.9%) were in RULA grand 5–6 group. (Table 3).

**Table 3:** Frequency of musculoskeletal complaints based on Final RULA score

RULA Score	3–4 N (%)	5–6 N (%)	> 7 N (%)	Chi-square value	*P* value
Organ					
Neck	3 (11.5)	19 (73.1)	4 (15.4)	3.49	0.17
Upper back	3 (15)	13 (65)	4 (20)	2.33	0.31
Lower back	4 (21.1)	13 (68.4)	2 (10.5)	0.13	0.93
Shoulder	1 (6.2)	11 (68.8)	4 (25)	5.86	0.06
Thigh	0 (0)	4 (11.5)	1 (11.5)	1.53	0.46
Knee	3 (25)	8 (66.7)	1 (8.3)	0.45	0.79
Wrist	5 (17.4)	14 (60.9)	4 (21.7)	1.47	0.47
Ankle	1 (20)	4 (80)	0 (0)	0.84	0.65

Table 4 show the presence of MSDs in participants based on their age, duration of occupation, mean weekly working hours and BMI. According to the result of this table the participants with pain in neck, knee and ankle were statistically significant older than their counterparts (*p* value = 0.02, *P* value = 0.01, *P* value< 0.001). In terms of duration of occupation, although subjects who complained of musculoskeletal pain had more work experience, but these differences were not statistically significant. In terms of BMI and mean weekly working hours, people with higher BMI and more weekly working hours only experienced statistically significant more ankle pain (*P* value = 0.01, *P* value = 0.008 respectively).

**Table 4:** Presence of musculoskeletal complaints in pathologists based on age, duration of occupation, the mean weekly working hours and BMI

		Age Mean (SD)	*P*-value	Duration of occupation Mean (SD)	*P*-value	Mean weekly working hours Mean (SD)	*P*-value	BMI Mean (SD)	*P*-value
**Neck**	Yes	38.30 (8.11)	**0.02**	7.88 (7.26)	0.05	47.76 (12.70)	0.07	24.30 (2)	0.05
	No	33.35 (5.18)		3.92 (2.58)		40.28 (12.06)		22.95 (2.23)	
**Upper Back**	Yes	38.35 (8.34)	**0.1**	7.90 (7.83)	0.1	52.10 (10.39)	0.001	24.42 (1.99)	0.08
	No	24.80 (6.37)		5.10 (3.99)		38.20 (11.35)		23.24 (2.20)	
**Lower Back**	Yes	38.36 (8.70)	**0.1**	8.52 (7.70)	0.05	45.94 (12.32)	0.7	23.82 (2.37)	0.9
	No	34.95 (6.08)		4.66 (4.06)		44.42 (13.5)		23.84 (2)	
**Shoulder**	Yes	38.12 (6.76)	0.2	6.62 (4.91)	0.9	46.25 (16.81)	**0.6**	24.51 (1.44)	0.1
	No	38.54 (7.99)		6.41 (7.18)		44.41 (9.69)		23.37 (2.45)	
**Thigh**	Yes	37.6 (4.82)	0.7	6 (1.87)	0.6	32.4 (3.28)	**0.00**	23.83 (1.13)	0.9
	No	36.42 (7.89)		6.57 (6.71)		46.97 (12.67)		23.83 (1.13)	
**Knee**	Yes	39.58 (10.36)	**0.01**	9.83 (9.13)	0.1	45.83 (13.34)	0.8	23.87 (2.47)	0.9
	No	35.28 (5.72)		5.07 (4.03)		44.85 (12.86)		23.81 (2.06)	
**Wrist**	Yes	37.34 (8.72)	0.4	7.21 (7.50)	0.3	49.39 (12.71)	**0.01**	24.24 (2.04)	0.07
	No	35.52 (5.65)		5.52 (4.18)		39.41 (10.92)		23.13 (2.18)	
**Ankle**	Yes	46.20 (9.87)	**0.00**	14.8 (10.54)	0.1	53.40 (3.13)	**0.008**	26.06 (1.13)	0.01*
	No	35.20 (6.25)		5.31 (4.56)		43.17 (13.29)		23.51 (2.08)	

In multivariate analysis, using logistic regression, the results showed that age and final RULA score had independent effect on neck pain (OR = 1.15, *P* value = 0.03 and OR = 2.50, *P* value = 0.04). In addition, BMI and final RULA score were independent variables in developing pain in upper back (OR = 1.47, *P* value = 0.03 and OR = 3.01, *P* value = 0.01). (Table 5).

**Table 5:** Factors significantly associated with MSDs in past 12 month based on logistic regression

Dependent variable	Independent variable	Crude odd’s ratio	Adjusted odd’s ratio	95% (CI)^a^	***P*** value
**Neck pain**	Age	1.11	1.15	1.01–1.31	0.03
	Final RULA score	1.89	2.50	1.02–6.07	0.04
**Upper back pain**	BMI	1.32	1.47	1.02–2.11	0.03
	Final RULA score	2.43	3.01	1.23–7.39	0.01

^a^Confidence interval

Age, BMI, mean weekly working hours and final RULA score were entered in model

In this study the chi-square test did not show statistically significant association between gender, dominant hand, smoking and presence of heart disease and developing pain and discomforts in neck, upper back, lower back, shoulder, thigh, knee, wrist and ankle.

## Discussion

The aim of this study was to determine the relationship between musculoskeletal complaints and working postures among the pathology specialists working at the laboratories affiliated to the Mashhad University of Medical Sciences. The mean age of the subjects was 36.57 years.

The highest incidence of MSDs among the subjects was in the neck (65%), wrist (57.5%) and upper back (50%). The MSDs were also observed in other part of the body like lower back (47.5%), shoulder (40%), knee (30%), ankle (12.5%) and thigh (12.5%), respectively. This situation may be due to inappropriate design of workstations. Working in laboratory station such as office jobs, often require a static position in the body due to their occupational status and sitting down on the chair over a long period of time, which has recently been reported as a major risk factor for the neck pain [14]. Ortiz-Hernandez et al. also showed an increase in the risk of developing MSDs in computer-related staff [15]. They demonstrated the prolonged use of the mouse, sitting so long, poor postures and psychological factors as major risk factors in the increased prevalence of MSDs. Bergqvist et al. reported the effective role of ergonomic factors, such as static postures, awkward hand postures, improper seat armchairs, repetitive movements, inappropriate placement of monitor and keyboard, in the prevalence of MSDs [16]. Among the pathologists, the posture while working with a microscope, computer and other devices make a situation where they are forced to put their neck and waist in an inappropriate condition, and the awkward design of workstations is exacerbating this issue.

Falaki et al. conducted a descriptive cross-sectional study reported that the incidence of neck pain was higher compared with the other body organs [11]. In the descriptive cross-sectional study in Iran, the highest prevalence of WMSDs within 12 months was reported in the neck (33.3%), followed by the shoulder and the elbow pain that was 21.6% [12]. Maulik et al. assessed the prevalence of musculoskeletal symptoms among the medical diagnosis laboratory technician in India. Overall incidence of MSDs reported by technicians was 73.3% in the trunk, knees and ankles. There was also a significant difference between the mean scores before and after working shift in the neck, waist and knees [17].

Regarding the studies in other similar occupations, Nokhostin et al. examined musculoskeletal disorders and its complications among dentists. They showed that 67.5% of dentists suffered from physical disorders including neck (51.8%), wrist (92.9%) and elbows (11.11%) and shoulder (7.4%) discomforts [18]. In a study of Rafeemanesh et al. the prevalence of MSDs in different parts of the body in dentists was obtained in the neck (75.9%), shoulders (58.6%), upper back (56.9%), lower back (48.3%) and wrists (44.8%) [19]. In a study by Juul-Kristensen et al. on 1428 office workers, it was found that the MSDs are high in similar occupations and are the highest in the neck, back and shoulders [20]. Kristensen et al. also showed that the incidence of these discomforts in office personnel in the neck, waist and shoulder areas were higher than in other parts of the body. In the studies of Szeto et al. on surgeons and Kumar et al. on dentists, the neck has had the highest MSDs [21, 22]. The results of all mentioned studies are in line with the findings of this study, indicating a high prevalence of MSDs in the neck and waist. A slight difference in the prevalence can be attributed to the nature of the work in different studies. In general, undoubtedly, the high prevalence of MSDs among the pathologists in the present study indicates the harmfulness of the working conditions and environment in this profession and their awkward physical postures during work.

In this study, the mean duration of occupation was 6.50 ± 6.30 years and the mean weekly working hours was 45.15 ± 12.84 h. The interaction between the long-term sitting position and the awkward workplace conditions may cause long-term static contraction of the muscles, hereby increasing pressure on the intervertebral discs, inducing muscle tension on the ligaments and muscles, reducing tissue flexibility and changing in the spine curvature. Therefore, the present study also found a high prevalence of MSDs in the upper back and the lower back areas. Meanwhile, the results demonstrated a significant relationship between the mean duration of occupation and the neck and lower back pain. There was also a significant relationship between the mean weekly working hours in the subjects and the discomfort in the thigh, upper back, wrists and ankles. In this case, the research has also shown that increasing daily work hours is associated with the higher prevalence of MSDs confirming the results of our study [23].

The mean age of participants was higher in people with discomfort in neck, knee and ankle. Similar to our results in the study of Nokhostin et al., a significant association was also observed between the age and the presence of MSDs [18]. The mean BMI of the physicians was 23.83 ± 2.16 kg/m^2^. In line with the results of other studies, BMI had statistically significant association with ankle pain [24–26]. In this study the pathologists with the higher mean working hours in week, complained of more pain in thigh, wrist and ankle. Different studies have shown that the association between long working time and development of occupational injuries and musculoskeletal disorders [27, 28]. According to the literature, the risk for musculoskeletal disorder increase in health care workers who had non-standard working time, including long working schedule, rotating or night shifts [29–31]. The results of the study demonstrated marginal significant relationship between work experience and MSD in neck and lower back. This result is consistent with some other studies [32–34]. Maybe, work experience modifies their life style and increase their knowledge about better ergonomic position and prevention of musculoskeletal injuries due to workplace conditions. Also, they develop some coping strategies like regular exercise, situational conformity and adjustment in work style. In addition, more experienced professional maybe more dedicated to supervisory and administrative work slides.

According to other examinations of the present study, the mean RULA grand scores was found to be 3 or 4 in 8 (20.0%), 5 or 6 in 27 (67.5%), and 7+ in 5 (12.5%). Accordingly, it can be claimed that the majority of the pathology specialists in our study were at least in third ergonomic level and it shows that most of them were in high risk of MSDs. Meanwhile, about 80% of cases were at least in 6 score and it indicates severe awkward posture of the body and thereby the abnormal ergonomics in this occupation. In the study of Maulik et al. the final RULA score was obtained to be 6 ± 1.02, emphasizing poor workplace condition among medical laboratory technicians. Their study showed that the prevalence of MSDs is high among medical laboratory technicians, and intervention of administrative and engineering controls can dramatically attenuate ergonomic risks, consistent with the results of our study [17]. Therefore, all pathologists present in our study should prioritize corrective actions as soon as possible.

A significant relationship between the mean RULA grand score and MSDs at upper back (*P* = 0.02) and shoulder (*P* = 0.02) during the last 12 months was noted. Also mean RULA score was higher in individuals with upper back and shoulder pain. There was also a significant relationship between the mean RULA *B* and neck pain (*P* = 0.02) and back pain (*P* = 0.05). A significant relationship was also seen between the mean RULA *A* and upper back and wrist pain (*P* = 0.003), indicating poor workstation design resulting in abnormal posture. Falaki et al. observed a significant relationship between awkward posture and pain in the body organs and finally showed high prevalence of MSDs in the medical laboratory personnel [11]. Unfit posture of these staff is expressed as the main cause of these disorders and corrective ergonomic actions are needed to reconstruct the workstations in laboratories. In another study on dentists, the analysis of work using REBA showed that 89.6% of the cases in group *A* and 79.3% of the cases in group *B* had a score of 4 that shows an awkward posture in this occupation [19]. Florian Rudalf Fritzsche et al. also emphasized the need for clinical care and ergonomic interventions in the workplace among the population of pathologists in Switzerland [35].

Among all pathologists of Mashhad University of Medical sciences, the highest prevalence of MSDs was observed in the neck, the wrists, the upper back and the lower back. These results will help to improve the working conditions, corrective actions, and paying attention to the risk factors of these areas. Some of the most important reasons for high RULA score among pathologists can be attributed to 1) the poor design of the workstation in our university 2) the awkward setting of the desk containing tools and gadgets, the impossibility of adjusting the seat height 3) non-compliance with ergonomic principles by the pathologist due to lack of proper training in this field and also huge workload. Therefore, monitoring and supervision the ergonomic principles in the workplace such as the laboratory will reduce these issues. Therefore, reducing working hours or increasing the rest time, conducting tests periodically, increasing accuracy in the correct positioning (such as allowing to adjust the height of the work desk, microscope, computer, and making available and standardizing other equipment) can be effective in the prevention of MSDs among the pathologists with high workloads.

Some of the limitations of this study were the small sample size and the lack of cooperation of some physicians (according to the self-report nature of the Nordic questionnaire). The psychological conditions in the workplace, occupational stress, and other external factors may also affect the outcomes of our project, which have not been addressed in this study. However, this study was the first one in our region was done on all pathologists who worked in one of the largest and equipped university of Iran. In addition, this study was conducted as a dissertation assistant of occupational medicine and with close supervision of her, and we have full confidence in the accurate filling in of questionnaires by participants, careful assessment of the work environment of by an occupational medicine specialist and the accuracy of the study results.

## Conclusion

The results of this study show high prevalence of musculoskeletal disorders and awkward working postures among the pathologists. Therefore, it is essential to perform ergonomic corrective actions in order to improve the physical conditions of their working environment and prevent work-related musculoskeletal disorders.

## Data Availability

The data that support the findings of this study are available from corresponding author but restrictions apply to the availability of these data, which were used under license for the current study, and so are not publicly available. Data are however available from the authors upon reasonable request and with permission of Mashhad University of Medical Sciences.
